# Transcriptome Analysis Reveals the Genes Related to Water-Melon Fruit Expansion under Low-Light Stress

**DOI:** 10.3390/plants12040935

**Published:** 2023-02-18

**Authors:** Wenrui Gao, Fuchun She, Yanjun Sun, Bing Han, Xiansheng Wang, Gang Xu

**Affiliations:** 1 Institute of Vegetable Crop, Jiangsu Province Academy of Agricultural Sciences, Nanjing 210014, China; 2 Jiangsu Key Laboratory for Horticultural Crop Genetic Improvement, Nanjing 210014, China; 3 School of Horticulture, Anhui Agricultural University, Hefei 230036, China; 4 Institute of Germplasm Resources and Biotechnology, Jiangsu Academy of Agricultural Sciences, Nanjing 210014, China; 5 Nanjing Station for DUS Testing Center of New Varieties of Plants of MARA, Nanjing 210014, China

**Keywords:** watermelon, low-light stress, fruit expansion, RNA-seq, differentially expressed genes, transcription factors

## Abstract

Watermelon is one of people’s favorite fruits globally. Fruit size is one of the important characteristics of fruit quality. Low light can seriously affect fruit development, but there have been no reports concerning molecular mechanism analysis in watermelons involved in fruit expansion under low-light stress. To understand this mechanism, the comparative transcriptomic file of watermelon fruit flesh at four different developmental stages under different light levels was studied. The results showed that the fruit size and content of soluble sugar and amino acids at low-light stress significantly decreased compared to the control. In addition, 0–15 DAP was the rapid expansion period of watermelon fruit affected by shading. In total, 8837 differentially expressed genes (DEGs) were identified and 55 DEGs were found to play a role in the four different early fruit development stages. We also found that genes related to oxidation-reduction, secondary metabolites, carbohydrate and amino acid metabolism and transcriptional regulation played a key role in watermelon fruit expansion under low-light stress. This study provides a foundation to investigate the functions of low-light stress-responsive genes and the molecular mechanism of the effects of low-light stress on watermelon fruit expansion.

## 1. Introduction

Fruit setting, cell division and cell expansion also play a crucial role in fruit development. These processes of growth largely determine the initiation and final form of fruit growth. Cell cycle-related genes play an important role in cell division and nuclear replication. Some enzyme-coding genes involved in cell wall extension play an important role in cell enlargement and maturation [1,2].

Light is essential for plant growth, development and productivity. Low light unbalances carbon assimilation, resulting in the inhibition of physiological attributes, morphology parameters and yields [3]. Many studies have shown that low light can seriously affect fruit development [4,5]. Shading treatments have been shown to significantly decrease photosynthesis, total nitrogen (T-N) of the stems and roots and the fruits and yields of strawberry plants. At the same time, the lower yield was due to the decreased photosynthesis of plants under low light [6]. The reduced growth of apple fruit in shade treatment was because of reduction in cell production and expansion. Moreover, shading results in coordinated changes in the expression of carbohydrate metabolism-related genes, key transcription factors related to fruit growth and genes associated with cell production and expansion [7]. During the early peach developmental stage, the formation of starch grains was inhibited and fewer photoassimilates were translocated from source leaves to fruit sinks [8]. After shading, the imported assimilates of the melon were reduced in number and galactosyl-sucrose oligosaccharide unloading was inhibited. In addition, the activity of key enzymes related to sucrose synthesis changed after shading, which affected sucrose content in melon fruit [9]. Crosstalk between the flavonoid biosynthetic genes and the involvement of key transcription factors such as McMYB4, McMYB7, McMYB10 and McMYB16 in the regulation of the ratio of anthocyanins and flavanols accounted for the different coloration of the fruit peel and softening of the flesh under shade conditions [10].

Watermelon (*Citrulluslanatus*) is a highly popular and economic horticultural crop. Approximately 3.48 million hectares were planted with watermelon in 2014 all over the world, making it among the top five most consumed fresh fruits, and China is the largest watermelon producer in the world. The watermelon plant produces large, edible fruit, the flesh of which is about 91% water by weight, and is a rich source of bioavailable compounds including lycopene and other carotenoids, vitamins A and C, and the non-essential amino acid L-citrulline, and is about 6% sugar by weight [11]. The size and weight of watermelon fruit are important aspects in agriculture and important directions of breeding research. In addition, fruit size is not only the important factor of yield, but also strongly associated with the watermelon’s commercial value. The size of fruit is regulated by internal heredity and environmental factors. Watermelon is a photophilic plant and often suffers from low-light stress in protective cultivation in early spring and autumn [12]. Research has shown that watermelon fruit size is significantly decreased as the planting density is increased, whereas the soluble solids content of the fruits is affected little. The fruit size of the watermelon is closely related to both the total solar radiation and the photosynthetic production per plant [13]. Our previous studies also show that the overcast and rainy climate in early spring and autumn leads to weak light in the facility, which seriously affects the growth of watermelon plants and the expansion of fruit. However, there have been no reports concerning transcriptome analysis in watermelon involved in fruit expansion under low-light stress.

Therefore, in this study, natural full light (CK) was recruited as a control to study the mechanism of watermelon fruit enlargement in response to low light (LL). We first analyzed the dynamic changes of fruit development under different light conditions. Then, the transcriptome of the watermelon fruit at four stages of development under low light and CK were analyzed using RNA-seq technology. We study and define the differential expression of genes of watermelon fruit at four different early fruit development stages. Meanwhile, gene annotation, GO classification, KEGG metabolic pathway, etc., were studied. Our hypothesis is that (1) the number and type of genes would differ in two treatments at different stages, and (2) that genes might play important roles during the process of fruit expansion under low light. Our aim is to find key genes and metabolic pathways that control fruit enlargement in the early fruit development stage and the molecular mechanism of low light regulation of the fruit expansion of watermelon. The outcome provides an important theoretical basis for the regulation of watermelon fruit size and fruit development.

## 2. Results and Discussion

### 2.1. Response of Watermelon Fruits Morphological and Physiological Dynamic Changes to Low-Light Stress

The early stages of fruit development, including fruit set and exponential growth, are clearly essential for all fruit. Early fruit development is typified by phases of cell division and expansion, which are critical determinants of size and yield. So far, most of studies, including transcriptomic studies, have focused on late development, or a broad range of developmental stages, with only a few studies focused on early fruit development stages [14].

**Figure 1:**
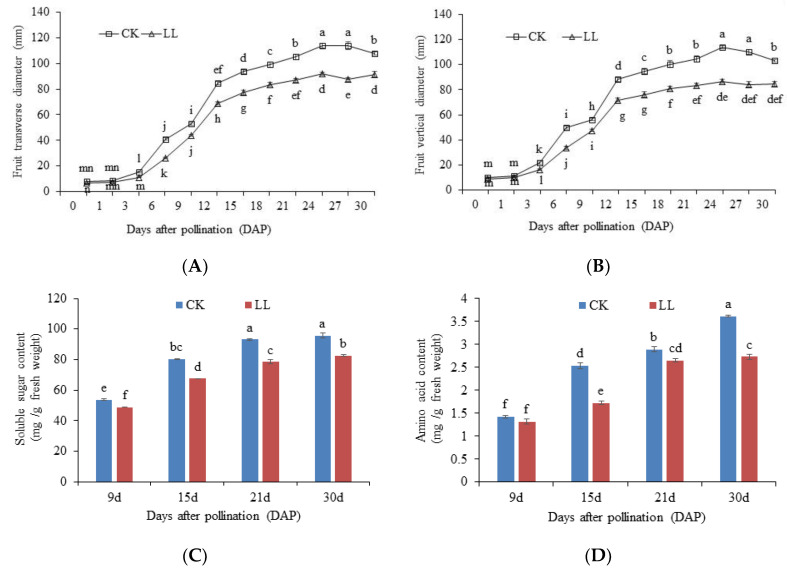
Watermelon fruit dynamic development under low-light stress. (**A**) Fruit vertical diameter. (**B**) Fruit transverse diameter. (**C**) Fruit soluble sugar content. (**D**) Fruit amino acid content. Error bars represent standard errors. Different letters (a, b, c, d, e, f) indicate significant statistical differences (*p* < 0.05).

In order to clarify the key period of watermelon fruit enlargement affected by low light, watermelon fruits’ vertical and transverse diameters at different time points after pollination were studied (Figure 1A,B). The results showed that the fruits’ vertical and transverse diameter of CK and LL increased sharply in 0–15 DAP, and then changed smoothly. The fruits’ vertical and transverse diameter of CK in 0–30 days were significantly larger than LL. This also indicated that 0–15 DAP was the rapid expansion period of watermelon fruit enlargement affected by low light intensity. Based on this result, we compared and analyzed the transcriptome of watermelon fruit at 0–15 DAP under LL and CK.

Sugar can provide metabolic energy for plants and act as substrates and signaling molecules in various metabolic pathways, and could provide carbon skeletons for the synthesis of amino acids and nucleotides [15]. Amino acids are mainly nitrogen-based metabolites that affect watermelon quality. During the increase in pollination days, the soluble sugar content and amino acid content of the LL group and the CK group showed a continuous upward trend, and the soluble sugar content and amino acid content of the LL group were significantly lower than the control group (*p* < 0.05), except that the amino acid at 9 DAP between the LL group and the control group was not significant (Figure 1C,D). Low-light stress reduces the photosynthetic performances of the plants, and the transportation of photosynthetic products from leaves to fruits are also affected [6,9], which ultimately leads to the reduction in the sugar content in fruits. Similar research results have been reported on melon [16], strawberry [6] and jujube [17]. Nitrogen metabolism was disturbed by shading, which induced the decrease in dry matter accumulation, ultimately resulting in the failure of watermelon fruits to expand normally [18]. This means that low-light stress affects the carbon and nitrogen metabolism of watermelon fruit, hindering the formation and transport of substances when watermelon fruit expands under low-light stress, thus making it difficult for watermelon fruit to expand normally.

### 2.2. Overview of Sequencing and Transcriptome Assembly

In order to explore the transcriptome of watermelon fruit expansion under low-light stress more comprehensively, we established 24 cDNA libraries and carried out RNA-seq, and 545.71 million raw reads were generated (Supplementary Table S1). In total, 527.59 million (96.68% of the raw reads) clean reads (approximately 158.28 Gb clean data) were obtained. On average, 21.98 million clean reads (6.59 Gb clean data) were obtained from each sample. The Q30 percentages ranged from 90.89% to 94.56%, and the average GC percentage was 44.32%. Among the 24 samples, 95.76–96.55% of the clean reads were mapped to the reference genome, and 89.77–91.36% of clean reads were uniquely mapped (Supplementary Table S1).

Analysis of saturation curves of 24 cDNA libraries (genes with FPKM ≥ 0.01) reveals that the gene coverage began to become saturated when clean reads exceeded 10 million (Supplementary Figure S1a). The average clean reads were 21.98 million, which exceeded the saturation threshold, indicating that the sequencing depth was enough for transcriptome studies. The sequencing reads of 24 samples were uniformly distributed from 5′ to 3′ of genes (Figure S1b).

In all, 27,063 unigenes were generated through cufflinks program, including 23,800 genes aligned to the watermelon reference genome and 3623 new genes. Among them, 82.70% (21,116 mapped unigenes and 1263 new genes) of genes were successfully annotated in at least one of seven databases, and only 5.73% (1550) of genes annotated in all databases (Table 1).

### 2.3. Identification of Differentially Expressed Genes (DEGs) in Watermelon Fruit

**Figure 2:**
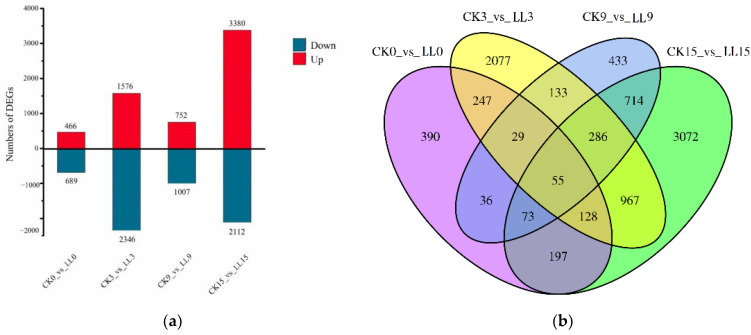
The numbers of up- and down-regulated differentially expressed genes (DEGs) between low light and control under different developments (**a**), Venn diagram of the differentially expressed genes (DEGs) between low light and control (**b**).

Compared with control, 8837 genes were differentially expressed in watermelon fruits under low-light stress using the criteria of FDR ≤ 0.01 and |log_2_ fold change| ≥ 1. There were 466, 1576, 752 and 3380 up-regulated genes and 689, 2346, 1007 and 2112 down-regulated genes after 0, 3, 9 and 15 DAP of low-light stress, respectively (Figure 2a). The numbers of up- and down-regulated genes were similar. At 0 DAP under low-light stress, alanine, aspartate and glutamate metabolism (ko00250) were the most significantly enriched KEGG entries in the down-regulated DEGs, while phenylpropanoid biosynthesis (ko00940) were the most in the up-regulated DEGs. At 3 DAP under low-light stress, genes involved in DNA replication (ko03030) were most enriched in down-regulated DEGs, while genes involved in plant hormone signal transduction (ko04075) were the most enriched in up-regulated DEGs. At 9 DAP of low-light stress, phenylpropanoid biosynthesis (ko00940) were the most significantly entries in down-regulated DEGs, while plant hormone signal transduction (ko04075) were the most entries in up-regulated DEGs. At 15 DAP, genes involved in the biosynthesis of secondary metabolites (ko01110) were most enriched in down-regulated DEGs, while genes involved in phenylpropanoid biosynthesis (ko00940) were enriched in up-regulated DEGs.

The results of Venn diagram showed that 390, 2077, 433 and 3072 DEGs were specifically at 0, 3, 9 and 15 DAP, respectively. 84 DEGs were all at 0, 3 and 9 DAP, 55 DEGs were shared in four time points (Figure 2b), suggesting their importance in the watermelon fruit expansion response to low-light stress. Among these 55 genes, three genes were up-regulated at four time points, suggesting their importance in watermelon fruit expansion response to low-light stress, while there was no gene down-regulated at four time points. The three genes included iron-sulfur binding oxidoreductase (*Cla008418*), two-component response regulator ARR12-like (*Cla016659*) and AT-hook motif nuclear-localized protein (*Cla006543*).

These 55 genes could be classified into five different categories according to KEGG pathway classification: metabolism, environmental information procession, genetic information processing, organismal systems and stress-related genes (genes with no KEGG pathway classification were assigned to this item). Most of the genes were classified into stress-related genes (Table 2).

Eighteen genes were divided into the metabolism category. Among these, plant glucan endo-1,3-*β*-glucosidases have been involved in diverse physiological and developmental processes including microsporogenesis, pollen germination, fertilization, response to wounding and cell division [16]. In our study, glucan endo-1,3-*β*-glucosidases was up-regulated by low-light stress, except for 9 DAP. Plant pyruvate decarboxylases (PDC) catalyze the decarboxylation of pyruvate to form acetaldehyde and CO_2_, and PDC derives the fermentation pathway involved in energy and material metabolism, responding to development, biotic and abiotic stresses [19]. PDC was down-regulated by low-light stress (except for 9 DAP) of watermelon fruit in our study, it may be a way to reduce the energy supply to fruit development under low-light stress. Through generating various intermediate products (such as reductant and pyruvate), glycolysis is the main pathway that supports respiration in plants. During the phosphorylation of Fru-6-P to Fru-1, 6-P2, PFP utilizes pyrophosphate (PPi) as an alternative phosphoryl donor in place of ATP, thereby providing an energy advantage to plants [20]. In our study, gene expression of *Cla004587* (except for 0 DAP) and *Cla017722* (except for 9 DAP) was down-regulated by low-light stress. This showed that low light can inhibit the energy supply of watermelon fruit development. Alcohol dehydrogenase (ADHs) gene expression produces enzymes that are not only active when plants are subjected to various stresses, but also during all developmental stages of plants under suitable growing conditions [21]. During the development of plant organs, ADH participates in glycolysis to provide energy for plants and regulate the metabolism of substances [22]. ADHs showed down-regulated patterns by low-light stress in this study (except for 9 DAP). This means that low light can affect the energy supply by inhibiting the expression of ADH during watermelon fruit development.

Three NRT1/PTR family (NPF) proteins can be detected in amino acid metabolism and were originally identified as nitrate or di/tri-peptide transporters. Recent studies revealed that this transporter family also transports the plant hormones and secondary metabolites (glucosinolates) [23]. The gene expression patterns of three NPF genes were up-regulated at 0 and 3 DAP and down-regulated at 9 and 15 DAP by low-light stress; this may lead to the changes in amino acid composition, hormone content and secondary metabolites during watermelon fruit development, which affected the size of watermelon fruit. Asparagine synthetase is able to assimilate ammonium in plants and it might be involved in N remobilization [24]. Asparagine synthetase was up-regulated at 0 DAP and down-regulated at 3, 9 and 15 DAP in our study. The reason may be that low-light stress can affect N metabolism. *Cla013862* encode acyl-[acyl-carrier-protein] desaturase (AAD) 6 (chloroplastic), and *Cla012276* and *Cla01235* encode cytochrome P450, which were classified into lipid metabolism. Interestingly, the expression patterns of AAD (chloroplastic) and cytochrome P450 are just opposite. Four genes (*Cla001815*, *Cla016032*, *Cla017208*, *Cla017207*) were classified into biosynthesis of other secondary metabolites. Except for copoletinglucosyltransferase-like, the expression patterns of the other three genes were first increased, then decreased and then increased again.

Seven genes involved in signal transduction, such as two-component response regulator ARR12-like (ARR12), ARR12 mediate the cytokinin regulated gene expression as myeloblastosis (MYB)-like transcription factors (TFs) and have both receiver and output domains. Type B Arabidopsis Response Regulators (ARRs) of Arabidopsis thaliana are transcription factors that act as positive regulators in the two-component cytokinin signaling pathway [25]. In our study, the expression level of ARR12 under low-light stress was higher than that of CK at four time points. The results showed that high level ARR12 affects watermelon fruit expansion and development by promoting the cytokinin signaling pathway under low-light stress. The AT-hook motif nuclear-localized protein (AHL) gene family, which encodes embryophyte-specific nuclear proteins with DNA binding activity, regulate gene expression and affect various developmental processes in plants, such as the modulation of GA and auxin biosynthesis, ABA-mediated stress growth regulation [26], etc. The AHL gene family regulates plant growth and development through forming DNA-protein and protein-protein homo−/hetero-trimericcomplex [27]. *Cla006543* (AT-hook motif nuclear-localized protein (AHL) 15) was also shown to exist at a higher level under low-light stress than CK at four time points. This means the AHL genes may play important roles through regulation of GA, ABA or auxin biosynthesis in watermelon expansion under low-light stress. *Cla023118* and *Cla009892* encode U-box domain-containing protein and mitochondrial translocator assembly and maintenance protein 41 homolog isoform X1, respectively, which are involved in genetic information processing. Two genes showed higher gene expression level at 0 and 3 DAP under low-light stress than CK, which mean that low light can affect watermelon fruit expansion through inhibiting genetic information processing.

Four genes are involved in environmental adaptation, which belong to organismal systems. Among them, two genes (*Cla007307*, *Cla007656*) encode WRKY transcription factor, one gene (*Cla018486*) encodes receptor-like protein kinase, and one gene (*Cla007979*) encodes transcription factor GLK2. Gene expression level of *Cla007307* (WRKY70) was down-regulated by low-light stress (except for 15 DAP), while *Cla007656* was up-regulated by low-light stress at 0 and 3 DAP and down-regulated by low-light stress at 9 and 15 DAP. Receptor-like protein kinase was down-regulated by low-light stress (except for 0 DAP) and transcription factor GLK2 was down-regulated at 0 and 3 DAP and up-regulated at 9 and 15 DAP by low-light stress. These results suggested that there is a complicated network for watermelon expansion and development under low-light stress.

A total of 24 genes were classified as stress-related genes. Among them, *Cla023376*, similar to early nodulin-like protein, was important for the transport of nutrients, solutes, amino acids or hormones and for major aspects of plant development [28]. In our study, nodulin-like protein 2 was down-regulated by low-light stress (except for 15 DAP), which showed that low light can inhibit the watermelon expansion and development by affecting the transportation of nutrients, amino acid, etc., to cause the fruit to be difficult to expand. Classical arabinogalactan protein 10-like (*Cla012888*) can play important roles in abiotic stress resistant, such as cold stress [29], and was up-regulated by low-light stress (except for 9 DAP). *Cla014570*, encoding dehydrins, belongs to the late embryogenesis abundant (LEA) protein family and was involved in responses to multiple abiotic stresses, such as cold and drought stress [30]. In our study, dehydrins were up-regulated by low-light stress (except for 0 DAP). Two genes (*Cla021693*, *Cla011361*) were both sugar transporters, which play important roles in plant growth and development, as well as biotic and abiotic stresses [31]. In addition, *Cla021693* (sugar transporter ERD6-like 16 isoform X2) was down-regulated by low-light stress (except for 0 DAP), while *Cla011361* (bidirectional sugar transporter SWEET4-like) was down-regulated at 0 and 9 DAP and up-regulated at 3 and 15 DAP by low-light stress. This indicated that low light affected the sugar transport, which led to the difficulty of watermelon fruit expansion under low light. It was noteworthy that *Cla008418* (Iron-sulfur binding oxidoreductase) was up-regulated by low-light stress at four time points.

### 2.4. Hierachical Clustering Analysis

**Figure 3:**
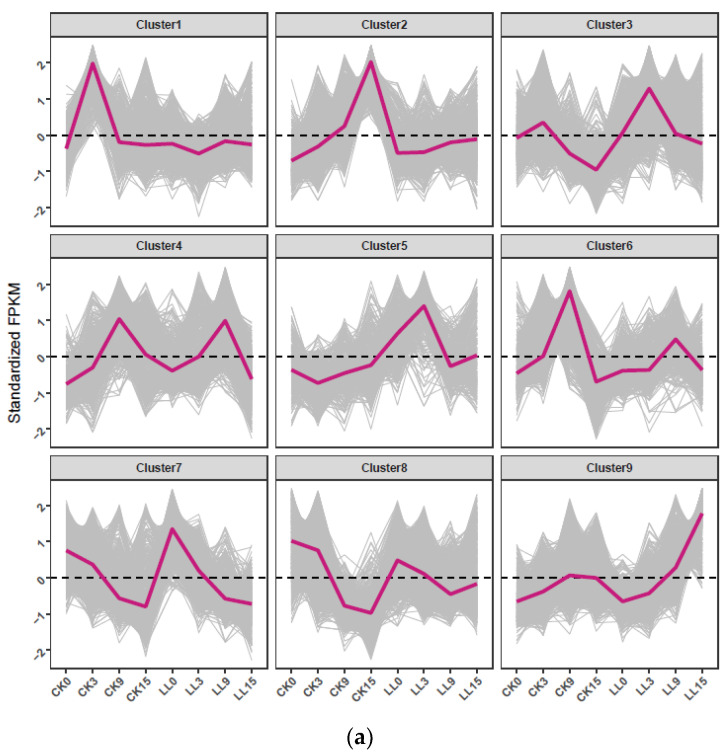

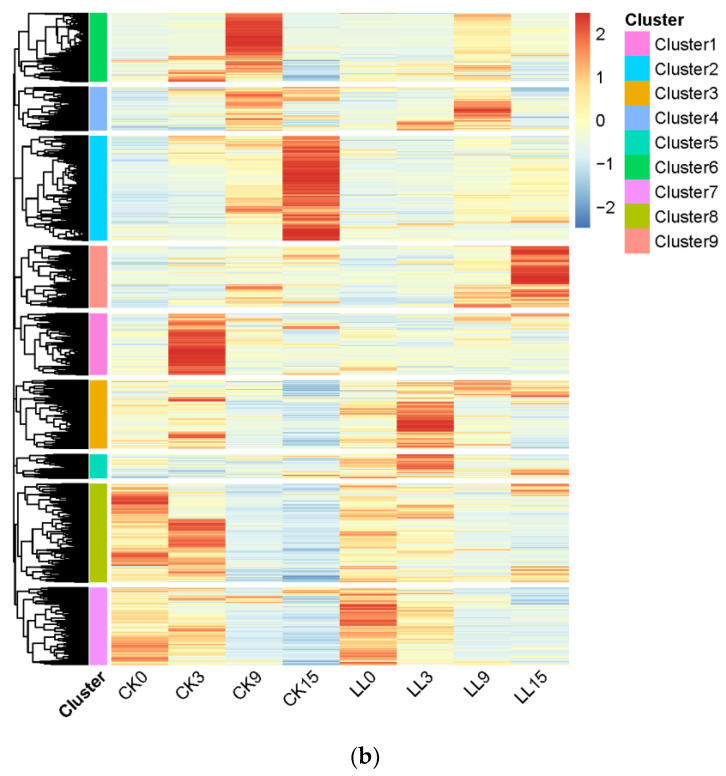
Clustering analysis of 8837 DEGs between CK and LL. (**a**). Hierarchical clustering of 8837 DEGs. (**b**). Expression patterns of the 8837 DEGs in the nine clusters.

Hierarchical clustering was performed for the 8837 DEGs based on similarity of gene expression (Figure 3). These DEGs were classified into nine gene clusters, and the genes’ expression pattern of CK and LL at different stages was different (Figure 3). Cluster 1 showed a relatively high expression level of CK compared with LL. The genes in cluster 1 mainly included genes encoding proteins related to replication, recombination and repair (*Cla003671*), posttranslational modification, protein turnover, chaperones (*Cla004677*, *Cla005777*), transcription (*Cla000971*, *Cla015165*) and carbohydrate transport and metabolism (*Cla011246*, *Cla020836*). The expression of the above genes was inhibited under low-light stress. The results showed that these genes may be related to watermelon fruit expansion under low-light stress. Meanwhile, cluster 3 and cluster 5 showed a relatively high transcript level in LL compared with CK. For example, these two clusters mainly included genes encoding proteins related to carbohydrate transport and metabolism (*Cla020563*, *Cla016546* and *Cla015950*), signal transduction mechanism (*Cla016899* and Cla016659), replication, recombination and repair (*Cla015651*, *Cla013640*), amino acid transport and metabolism (*Cla019133*, *Cla003285*) and transcription (*Cla007586*, *Cla016837*). Gene expression levels in cluster 7 and cluster 8 decreased with the development of fruit, but the gene expression pattern in cluster 2 and cluster 9 was contrary. The rest of the DEGs were clustered into cluster 4 and cluster 6.

GO enrichment analysis of the three clusters may be relevant to watermelon expansion under low-light stress. The results showed that the GO terms related to replication, recombination and repair, posttranslational modification, protein turnover, chaperones and transcription were highly enriched in cluster 1, such as GO:0006261 (DNA-dependent DNA replication), GO:0042138 (meiotic DNA double-strand break formation), GO:0048589 (developmental growth), GO:0003713 (transcription coactivator activity). In addition, we found that cluster 3 and 5 showed that GO:0007155 (cell adhesion) and GO:0045010 (actin nucleation) were most highly enriched.

### 2.5. Weighted Correlation Network Analysis of Low-Light Stress Responsive Genes Related to Watermelon Fruit Expansion

Weighted gene coexpression network analysis (WGCNA) is used frequently to explore effectively the relationships between genes and phenotypes. WGCNA can also analyze the target genes at a network-level [32] in order to identify the low light responsive genes correlated with watermelon fruit expansion. In this study, 10 modules were identified from the RNA-seq data (Figure S2a).

The module eigengenes for 10 modules were correlated with different test indicators. Analysis of the module-trait relationship showed that the module ‘MElightpink4’ and ‘MElavenderblush1’ were highly correlated with watermelon fruit expansion under low-light stress. ‘MElightpink4’ module was positively correlated with FVD (cor = 0.88, P = 1 × 10^−8^), FTD (cor = 0.87, P = 3 × 10^−8^), SFW (cor = 0.81, P = 2 × 10^−8^), AA (cor = 0.88, P = 2 × 10^−8^) and SS (cor = 0.85, P = 2 × 10^−7^). ‘MElavenderblush1’ module was negatively correlated with FVD (cor = −0.95, P = 4 × 10^−13^), FTD (cor = −0.95, P = 3 × 10^−12^), SFW (cor = −0.78, P = 7 × 10^−6^), AA (cor = −0.89, P = 4 × 10^−9^) and SS (cor = −0.88, P = 1 × 10^−8^) (Figure S2b). The module ‘MElightpink4’ included 4059 genes mainly containing genes related to “posttranslational modification, protein turnover, chaperones”, “carbohydrate and amino acid transport and metabolism” and transcription. 5643 genes, mainly including genes encoding transcription, replication, recombination and repair and signal transduction mechanisms were identified in the module ‘MElavenderblush1’. The results were almost consistent with earlier hierarchical clustering analysis.

To further understand the mechanism of watermelon fruit expansion under low-light stress, the GO enrichment and KEGG pathway of genes in the three modules were analyzed. The unigenes in the ‘‘MElightpink4’ module were mainly enriched in aromatic amino acid family biosynthetic process (GO:0009073), proteolysis (GO:0006508), isopentenyl diphosphate biosynthetic process (GO:0019288), photorespiration (GO:0009853), ubiquitin-dependent protein catabolic process (GO:0006511) and glycolytic process (GO: 0006096). While the highly enriched terms of the ‘MElavenderblush1’ were associated with translation (GO:0006412) and embryo sac egg cell differentiation (GO:0009560). The most significantly entries in KEEG analysis of the ‘MElightpink4’ and ‘MElavenderblush1’ module were biosynthesis of secondary metabolites (Ko:map01110) and ribosome (Ko:map03010), respectively (Tables S2 and S3).

### 2.6. Functional Classification of DEGs

Through the WEGO database, the DEGs were classified into 14 categories for cellular components, 11 categories for molecular functions and 22 categories for biological processes. 9037 unigenes and 3217 DEGs were assigned GO terms and a hierarchical relationship picture is drawn in Figure S3. The DEGs involved in cell part (1187), cells (1179) and organelles (867) were the most abundant entries in cellular components. The two most common categories for molecular functions were catalytic activity (1794) and binding (1559). For biological processes, metabolic processes (2018) and cellular processes (1517) were the most two abundant catalogs.

**Figure 4:**
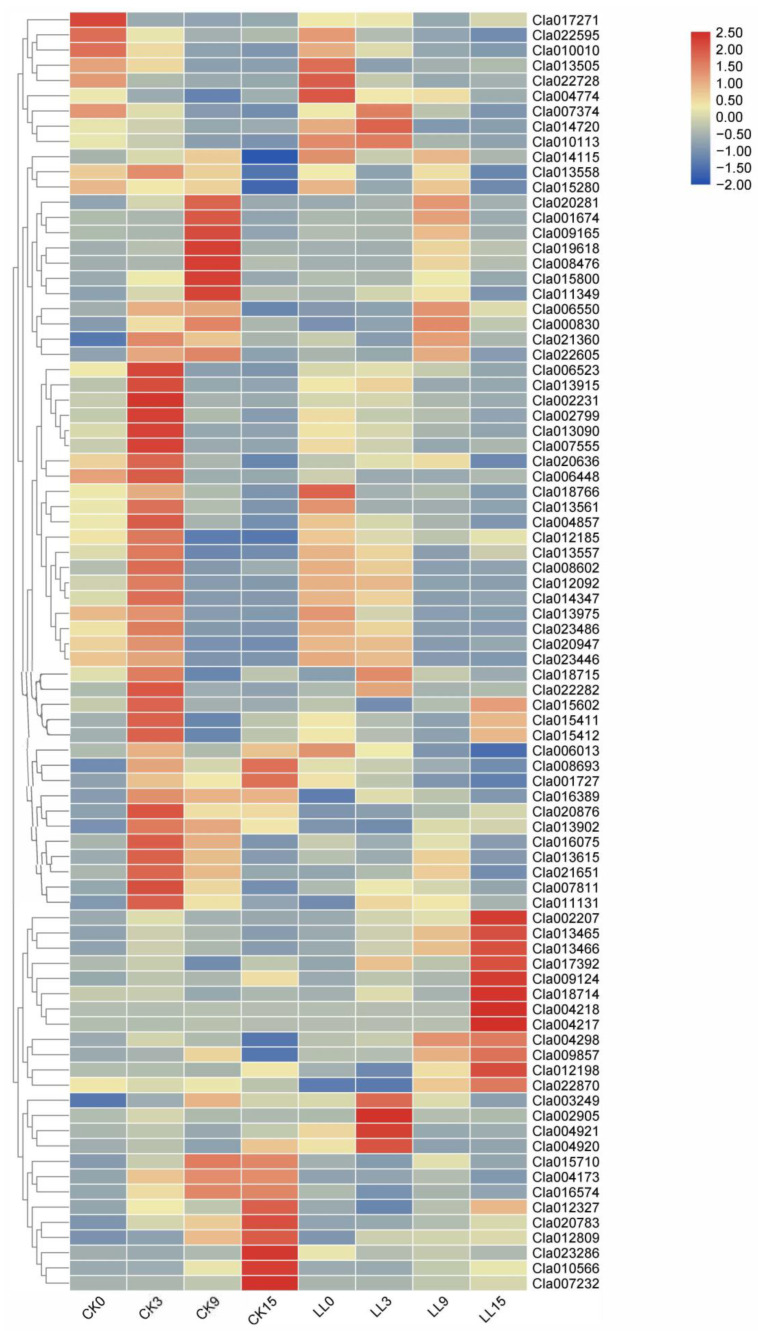
The raw FPKM values of the DEGs (with KOG classification was cell wall/membrane/envelop biogenesis and cell cycle control, cell division, chromosome partitioning) were first normalized by logarithmic method and a heat map was constructed to show the different expression profiles using the Heml software (version 1.0, http://hemi.biocuckoo.org/. accessed on 9 July 2020). *X*-axis, samples; *Y*-axis, differentially expressed gene names.

GO enrichment analyses of DEGs were also carried out by topGO. Oxidation-reduction process (GO: 0055114), salicylic acid-mediated signaling pathway (GO: 0009863) and cell wall organization (GO: 0071555), were the top three enriched in biological process (Table S4). Integral component of membrane (GO: 0016021), extracellular region (GO: 0005576), apoplast (GO: 0048046) and plant-type cell wall (GO: 0009505) were the top four enriched terms in cellular processes (Table S5). Cellulose synthase (UDP-forming) activity (GO: 0016760), oxidoreductase activity, acting on paired donors, with incorporation or reduction in molecular oxygen (GO: 0016705) and oxidoreductase activity (GO: 0016491) were the top three enriched terms in molecular functions (Table S6). It is not surprising that many of the DEGs identified in this study are involved in or associated with cell wall metabolism. Plant cell walls are dynamic structures that determine and maintain the size and the shape of the cells and act as a protective barrier, and they both resist the stresses generated by the hydrostatic pressure of the cells and have the ability to undergo the irreversible deformation associated with cell expansion [33]. In our study, gene expression patterns of the DEGs (with KOG classification was cell wall/membrane/envelop biogenesis and cell cycle control, cell division, chromosome partitioning) were analyzed (Figure 4). The results showed that the number of down-regulated genes was higher than that of the up-regulated genes at 0 DAP and 3 DAP, especially at 3 DAP, which means that genes related to cell wall and cell division were inhibited by low-light stress and low light affects cell division and replication at the initial stage of watermelon fruit pollination. The number of up-regulated genes was significantly more than that of down-regulated genes at 15 DAP, indicating that these genes can resist low-light stress in watermelon fruit development.

**Figure 5:**
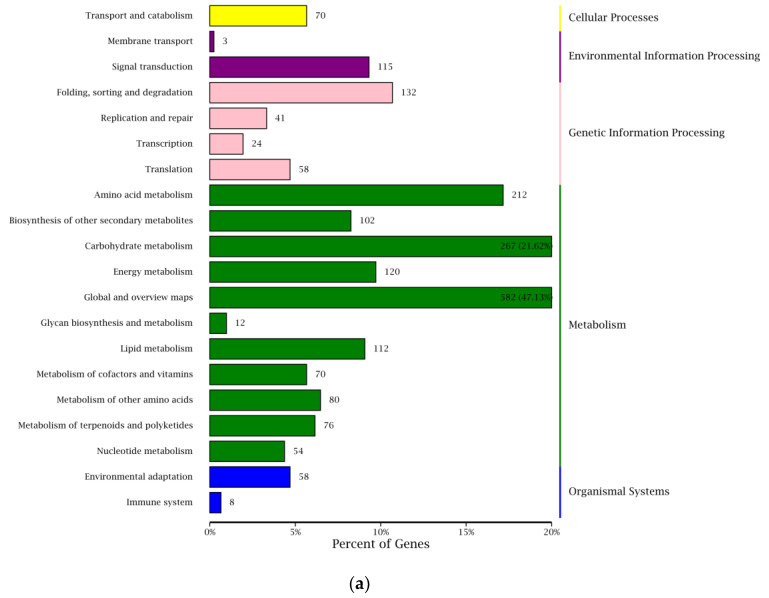

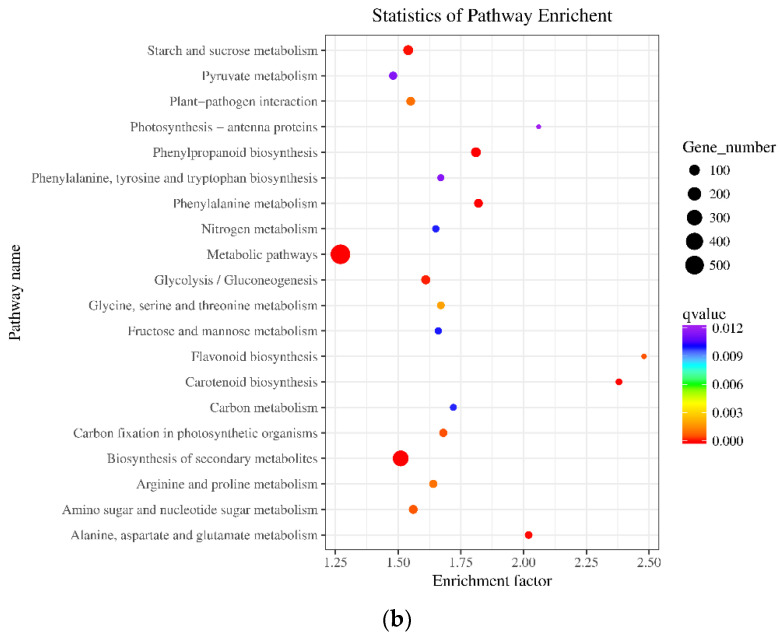
KEGG pathway enrichment analysis. (**a**) Statistic analysis of annotated genes in KEEG pathways, (**b**) scatterplot of KEEG pathway enrichment for DEGs.

Pathway enrichment analysis can identify the major biochemical and signal transduction pathways in which the DEGs were involved. As shown in Figure 5, biosynthesis of secondary metabolites (ko01110), phenylpropanoid biosynthesis (ko00940), phenylalanine metabolism (ko00360), carotenoid biosynthesis (ko00906), alanine, aspartate and glutamate metabolism (ko00250), starch and sucrose metabolism (ko00500), glycolysis/gluconeogenesis (ko00010), carbon fixation in photosynthetic organisms (ko00710), flavonoid biosynthesis (ko00941), amino sugar and nucleotide sugar metabolism (ko00520) were the ten most significantly entries.

As a substrate of protein biosynthesis, amino acid phenylalanine is necessary for the survival of all cells. Thus, the routing of newly synthesized phenylalanine into protein is a primary metabolic pathway. In plants, phenylalanine is also a substrate of phenylpropanoid metabolism [34]. Phenylpropanoids contribute to plant responses to biotic and abiotic stress. They are not only indicators of plant stress responses upon variation of light or mineral treatment, but also key mediators of the plant’s resistance towards pests. The general phenylpropanoid metabolism generates an enormous array of secondary metabolites based on the few intermediates of the shikimate pathway as the core unit [35]. Secondary metabolites play an important role in the adaptation of plants to the changing environment and in overcoming stress constraints. Carotenoids, including lycopene, and flavonoids are secondary metabolites in watermelon fruit, as well as in other fruits such as tomato, suggesting carotenoids and flavonoids are important to watermelon fruit expansion or development under low-light stress [36]. These results showed that these multiple pathways might contribute to fruit development and expansion under low-light stress within a complicated pathway network. In addition, the roles of oxidation-reduction, secondary metabolites, carbohydrate and amino acid metabolism are particularly critical.

### 2.7. Analysis of Transcription Factors (TFs) Involved in Watermelon Fruit Development and Response to Low-Light Stress

**Figure 6:**
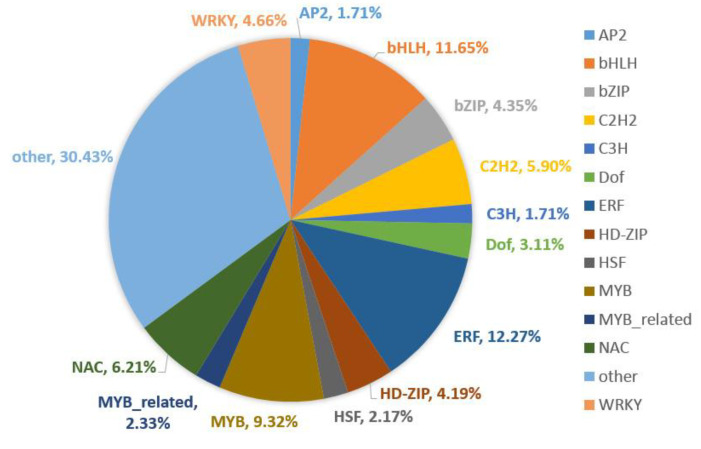
Distribution of differentially expressed TFs.

Transcription factors are essential for the regulation of gene expression through binding to specific *cis*-acting elements in their regulated genes. We found 644 TFs in DEGs, which can be classified into 53 TF families that were present in the plantTFcat database including ERF (12.27%), bHLH (11.65%), MYB (9.32%), NAC (6.21%), C2H2 (5.90%) and WRKY (4.66%) (Figure 6). Of these differentially expressed TFs, bHLH, ERF, NAC and WRKY are all the most abundant in the 0, 3, 9 and 15 DAP (Figure S4). This means that these four TFs were important for watermelon fruit expansion under low-light stress. ERF have been shown to be intimately connected to plant development, defense responses and stress signaling pathways. The development of a plant organ to a specific size and shape is controlled by cell proliferation and cell expansion. Differentiation of xylem elements involves cell expansion, secondary cell wall (SCW) deposition, etc. The study shows ERF139 as a transcriptional regulator of xylem cell expansion and secondary cell wall formation, and possibly in response to the osmotic changes of the cells [37]. Many studies showed that bHLH plays critical roles in plant growth and development, metabolic regulation, and response to environmental changes, such as fruit development [38] and drought and salt stress [39]. MYB plays a vital role in organ development by directly affecting cell wall structure and/or cytoplasmic growth or indirectly regulating through the ethylene and/or ABA signaling pathways [40]. As a major component of plant cell walls, lignin plays important roles in mechanical support, water transport, and stress responses. The study shows NAC was associated with fruit lignification by activating genes involved in lignin biosynthesis [41]. WRKY can also play significant roles in stress responses and cellular growth [42]. Some TFs from the same family were up-regulated while others were down-regulated (Figure S5). The results demonstrated that members of the same TF family may play different roles in fruit development under low-light stress, and the transcriptional network for watermelon fruit expansion under low-light stress is intricate and complex.

### 2.8. Validation of RNA-Seq Data by qRT-PCR Analysis

**Figure 7:**
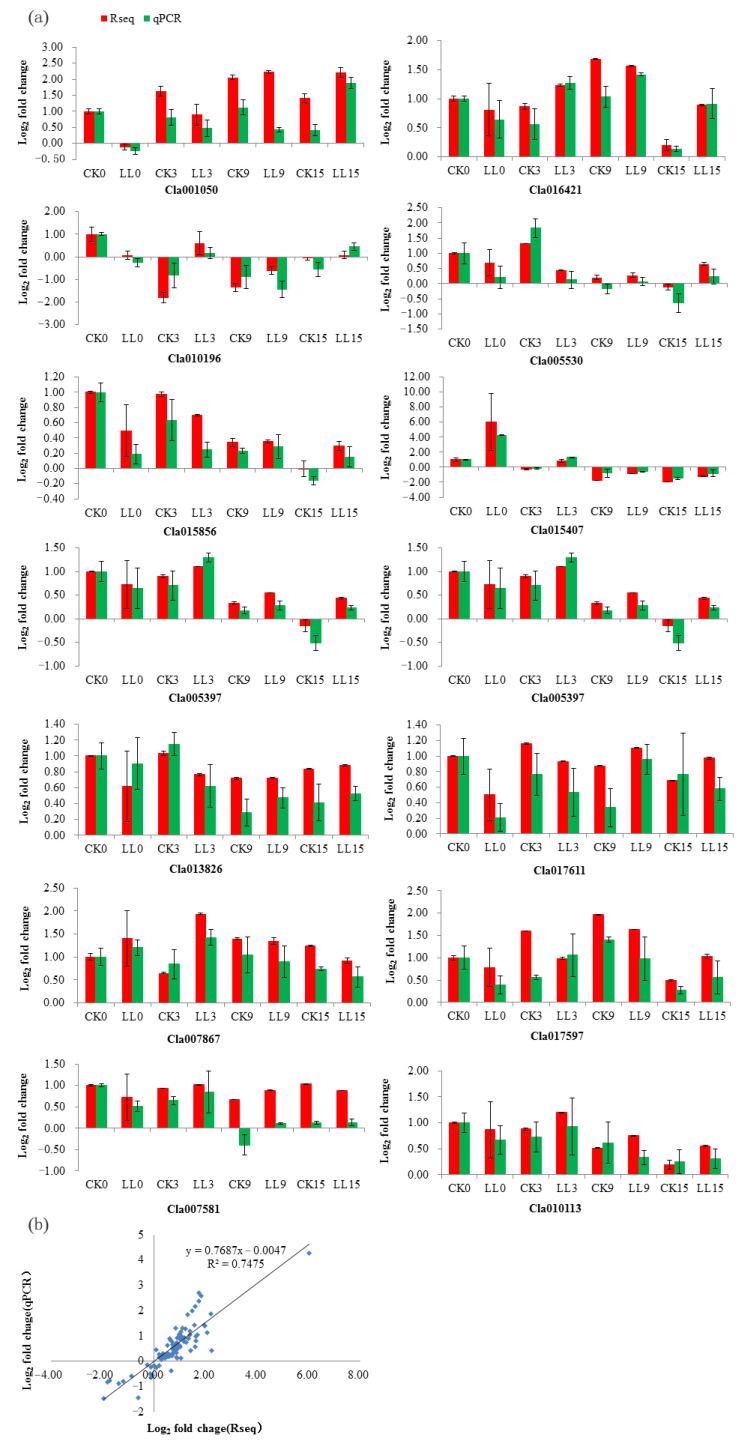
Verification of the expression of selected DEGs by qRT-PCR. (**a**) Log_2_ fold change of 14 genes under control and low-light stress at four developments of watermelon fruit. The bars represent the mean of three biological replicates ± standard errors. (**b**) Log_2_ fold change of RNA-seq (*X*-axis) and qRT-PCR (*Y*-axis).

To verify the RNA-seq data, 14 DEGs were selected randomly for qRT-PCR analysis (Figure 7a). In order to facilitate the comparison of the expression data between RNA-seq and qRT-PCR, the relative expression level was converted to log_2_ fold change. The qRT-PCR results showed a high consistency (linear regression equation y = 0.7687x − 0.0047, R² = 0.7475) with RNA-seq data (Figure 7b), indicating the high reliability of RNA-seq expression profile in this study.

## 3. Materials and Methods

### 3.1. Plant Materials

This research was performed in a steel frame plastic greenhouse with internal circulation fan, which can ensure the same temperature in the plastic greenhouse, at Luhe production base in Jiangsu Academy of Agricultural Sciences. Watermelon variety ‘Sumi No.8’ (Jiangsu Academy of Agricultural Sciences) was used as plant materials. ‘Sumi No.8’ is a new watermelon hybrid with early maturity and small fruit (an average single fruit weight is around 1.5 kg). The fruit flesh is yellow, and the fruit development period is about 30 d after pollination. Seeds were sown in plug tray (54 cm length, 27 cm width, 50 holes) filled with vegetable seedling commercial organic substrate (HengAoda Fertilizer Technology Co., Ltd., Lianyungang, China). The healthy and uniform size seedlings at the four-leaf stage were transplanted into the plastic pots (height, 26.5 cm; diameter, 31 cm) containing the organic culture substrate (cassava residue: peat: vermiculite = 2:1:1 in volume). Fertilizer, control of diseases and insects and other agronomy control was performed according to standard management practices.

Treatments were carried out 7 days before pollination. The low light (LL) groups of watermelon were grown under one layer of black sunshade net (Xinqiao agricultural screen Co., Ltd., Taizhou, China) and the light intensity of LL was 150–650 μmol quanta m^2^ s^−1^, while the control group (CK) was grown under natural full light (310–1300 μmol quanta m^2^ s^−1^). All flowers for each experiment were hand pollinated in the 9–10 node order, and only one fruit remained per plant. The flesh of fruit center was harvested at 0, 3, 9 and 15 days after flowering, and immediately frozen in liquid nitrogen and stored in a −80 °C freezer until use. At each sampling point, three fruits’ flesh were collected in one biological replicate, and three separate, biological replications of the fruits’ flesh were performed for RNA-seq.

### 3.2. Morphological and Physiological Index Measurements and Statistical Analysis

Prior to the harvests, fruit vertical and transverse diameter were measured at 0, 1, 3, 6, 9, 12, 15, 18, 21, 24, 27 and 30 DAP. Soluble sugar content and amino acid (AA) content were measured using a Plant Soluble Sugar Content Assay Kit (Suzhou Keming Biotechnology Co., Ltd. Suzhou, China) with 0.1 g samples of watermelon flesh at 9, 15, 21, 30 DAP.

### 3.3. cDNA Preparation and RNA Sequencing

The RNA of 24 samples was extracted using RNAprep Pure Plant Kit (TIAGEN, Beiing, China). After DNAse I treatment, mRNA was purified from the total RNA using the Oligotex mRNA Midi Kit (QIAGEN, Dusseldorf, Germany). The first cDNA strand was synthesized from purified mRNA fragments by reverse transcription with random hexamer adaptors (Invitrogen, California, MD, USA). Double strand cDNA was then synthesized using the SMART cDNA Library Construction kit (Clontech, Mountain View, CA, USA) according to the manufacturer’s instructions. The cDNA fragments with suitable lengths and insert sizes were selected by AMPure XP beads to construct cDNA libraries. The cDNA libraries’ quality was checked by Qubit2.0 and Agilent 2100 before they were sequenced using the IlluminaHiSeq 2500 platform, and the 150 bp paired-end reads were generated by Genepioneer Biotechnologies Co, Ltd. (Nanjing, China).

### 3.4. Transcriptomic Analysis

The raw sequence data have been submitted to the National Center for Biotechnology Information (NCBI) databases, for which the study accession was SRP243255. Clean reads were obtained by removing duplication sequences, adaptor sequences and low-quality reads (ambiguous sequences with ‘N’ percentage values ≤ 3 and the percentage of low-quality bases less than 3 is ≥50%) from raw reads. The cleaned reads were subsequently mapped to the watermelon reference genome (ftp://cucurbitgenomics.org/pub/cucurbit/genome/watermelon/97103/v1/watermelon_v1.genome.gz. accessed on 29 September 2018) by HISAT2 software (v2.1.0). Subsequently, the sequencing saturation, coverage analysis and the genomic distributions in CDS (exons), introns, and intergenic regions were analyzed.

### 3.5. Digital Gene Expression Tag Profiling and Identification of Differentially Expressed Genes (DEGs)

The expression of all genes was determined with FPKM (Fragments PerKilobase of exon per million fragments Mapped) values using the software Cufflinks software (v2.2.1) (http://cole-trapnell-lab.github.io/cufflinks, accessed on 29 September 2018). R package DESeq was used to identify DEGs by comparing the low-light stress and control at the same time point, with the false discovery rate (FDR) < 0.05 and |log_2_ fold change|≥ 1.

### 3.6. Gene Annotation and Functional Annotation and Analysis

All genes were compared against NR, GO, COG, KOG, Swiss-Prot, KO using BLASTn (v2.2.26) with an E-value cut off at 10^−5^ and searched against the Protein family (Pfam) database by hmmscan (v3.0) (https://www.ebi.ac.uk/Tools/hmmer/search/hmmscan/, accessed on 29 September 2018). Based on GO classification, all genes were analyzed for their functional characteristics and gene expression profiles under low-light stress and controlled conditions. GO enrichment analyses of DEGs were performed using singular enrichment analysis (SEA) method with *p* < 0.01 and FDR < 0.05 by topGO, and the watermelon genome was set as background. KOBAS (v2.0) (https://www.biostars.org/p/200126/) was employed in validation of gene expression (FDR < 0.05) for pathway enrichment analysis. All genes were BLAST against the Plant Transcription Factors Database (v3.0) (http://plntfdb.bio.uni-potsdam.de/v3.0/, accessed on 29 September 2018) with an E-value cut off at 10^−5^.

### 3.7. Weighted Correlation Network Analysis (WGCNA)

We built a correlation network using R package WGCNA. The adjacency matrix was created through calculating the pearson’s correlations. The pickSoftThreshold was used to calculate the soft thresholding power β. The topological overlap measure (TOM) was calculated by the adjacency matrix and TOM dissimilarity was used to build the dendrogram. The modules were detected as branches of the dendrogram using the dynamic tree-cut with a cut off height of 0.30 to merge the branches to final modules. The module eigengene (ME) value was calculated and used to estimate the association of modules with low-light stress responsive genes related to physiological index.

### 3.8. qRT-PCR Analysis

We chose 14 DEGs with different expression patterns for validation RNA-seq data by quantitative reverse transcription-polymerase chain reaction (qRT-PCR). *Actin* (*Cla007792*) was used as reference gene. The primers were designed using Primer 5.0. Primer sequences and gene annotations are listed in Table S7. QRT-PCR assays were conducted with three biological and three technical replicates. RNA for each sample was reverse-transcribed into first-strand cDNA using M-MLV reverse transcriptase (TaKaRa, Tokyo, Japan). QRT-PCR was conducted on an ABI 7300 real-time PCR system (Applied Biosystems, Foster City, CA, USA) using the SYBR Premix Ex Taq II reagent (Takara, Tokyo, Japan). QPCR conditions were set at 95 °C pre-denaturation for 3 min, 40 cycles of 95 °C for 15 s, 60 °C for 30 s, and 72 °C for 15 s. Gene expression levels were calculated by previous 2^−ΔΔCt^ method [43]. The correlation of RNA-seq with qRT-PCR analysis was calculated using SAS (v9.3) proccorr based on log_2_ fold changes.

## 4. Conclusions

In conclusion, this study confirmed that 0–15 DAP was the rapid expansion period of watermelon fruit under low-light stress and CK. Low light significantly inhibited fruit expansion, and the contents of soluble sugar and amino acids were also decreased. As far as we know, this work is the first study to provide comprehensive sequencing and DEG profiling data for a dynamic view of the transcriptomic variation in watermelon fruit development under low-light stress. In total, total 8837 DEGs were obtained in watermelon fruit flesh under low-light stress compared with the control, and 55 DEGs were shared among four stages of fruit development. At 0 DAP under low-light stress, alanine, aspartate and glutamate metabolism (ko00250) were the most significantly enriched KEGG entries in the down-regulated DEGs, while phenylpropanoid biosynthesis (ko00940) were the most enriched in the up-regulated DEGs. GO and KEGG analysis of the DEGs showed that these DEGs were mainly involved in oxidation-reduction, secondary metabolites, carbohydrate and amino acid metabolism, which indicated that the genes and pathways may be related to fruit expansion under low-light stress. In addition, the function and metabolic pathway of up-regulated and down-regulated DEGs at different time points were also different. At 3 DAP under low-light stress, genes involved in DNA replication (ko03030) were the most enriched in down-regulated DEGs, while genes involved in plant hormone signal transduction (ko04075) were the most enriched up-regulated DEGs. At 9 DAP of low-light stress, phenylpropanoid biosynthesis (ko00940) were the most significantly entries in down-regulated DEGs, while plant hormone signal transduction (ko04075) had the most entries in up-regulated DEGs. At 15 DAP, genes involved in the biosynthesis of secondary metabolites (ko01110) were most enriched in down-regulated DEGs, while genes involved in phenylpropanoid biosynthesis (ko00940) were enriched in up-regulated DEGs. Through WGCNA analysis, the modules of ‘MElightpink4’ and ‘MElavenderblush1’ were highly correlated with watermelon fruit expansion under low-light stress. The unigenes in the ‘MElightpink4’ module were mainly enriched in ubiquitin-dependent protein aromatic amino acid family biosynthetic process (GO:0009073), proteolysis (GO:0006508), isopentenyl diphosphate biosynthetic process (GO:0019288), photorespiration (GO:0009853), ubiquitin-dependent protein catabolic process (GO:0006511) and glycolytic process (GO: 0006096), while the highly enriched terms of the ‘MElavenderblush1’ were associated with translation (GO:0006412) and embryo sac egg cell differentiation (GO:0009560). The results were almost consistent with earlier hierarchical clustering analysis and physiological index analysis. Of differentially expressed TFs, bHLH, ERF, NAC and WRKY are all the most abundant at 0, 3, 9 and 15 DAP, which means that these four TFs were important for watermelon fruit expansion under low-light stress. Members from the same TF family may play different roles in fruit development under low-light stress, and the transcriptional network for watermelon fruit expansion under low-light stress is intricate and complex. Therefore, our study elucidated the key genes and pathways related to watermelon fruit expansion under low-light stress, which provided a theoretical basis and foundation for further research on fruit expansion under low-light stress.

## Figures and Tables

**Table 1:** Summary of the gene annotations in seven databases.

Annotation Database ^1^	Annotated_Number	Percentage
COG_Annotation	8060	36.02%
GO_Annotation	9037	40.38%
KEGG_Annotation	5156	23.04%
KOG_Annotation	11,715	52.35%
Pfam_Annotation	17,006	75.99%
Swissprot_Annotation	16,206	72.42%
nr_Annotation	22,343	99.84%
Annotated in all seven Database	1550	5.73%
Annotated in at least one Database	22,379	82.70%
All_Annotated	27,063	100.00%

^1^ Clusters of orthologous groups of proteins (COG) database, gene ontology (GO), KEGG ontology (KO), eukaryotic orthologous groups (KOG), protein family (Pfam), reviewed protein sequence database (Swiss-Prot), NCBI non-redundant protein database (NR).

**Table 2:** 55 DEGs that were shared at all four time points.

Gene ID	Gene Description	CK0_vs _LL0	CK3_vs _LL3	CK9_vs _LL9	CK15_vs _LL15
**Metabolism**					
**Carbohydrate metabolism**					
*Cla005337*	glucan endo-1,3-beta-glucosidase 13	Up	Up	Down	Up
*Cla018009*	pyruvate decarboxylase 1	Down	Down	Up	Down
*Cla004587*	pyrophosphate—fructose 6-phosphate 1-phosphotransferase subunit beta-like	Up	Down	Down	Down
*Cla017722*	fructose-bisphosphate aldolase cytoplasmic isozyme isoform X2	Down	Down	Up	Down
*Cla003920*	probable ribose-5-phosphate isomerase 1	Up	Up	Down	Down
*Cla015028*	alcohol dehydrogenase 1	Down	Down	Up	Down
**Amino acid metabolism**					
*Cla010147*	LOW QUALITY PROTEIN: protein NRT1/PTR FAMILY 2.13-like	Up	Up	Down	Down
*Cla010146*	protein NRT1/PTR FAMILY 2.13-like	Up	Up	Down	Down
*Cla019134*	protein NRT1/PTR FAMILY 2.11-like	Up	Up	Down	Down
*Cla013371*	asparagine synthetase [glutamine-hydrolyzing]	Down	Up	Up	Up
**Nucleotide metabolism**					
*Cla022500*	deoxyuridine 5&apos;-triphosphate nucleotidohydrolase	Up	Down	Down	Down
**Lipid metabolism**					
*Cla013862*	acyl-[acyl-carrier-protein] desaturase 6, chloroplastic	Down	Down	Up	Down
*Cla012276*	cytochrome P450 734A1-like	Up	Up	Down	Up
*Cla001235*	cytochrome P450 86A8-like	Up	Up	Down	Up
**Biosynthesis of other secondary metabolites**					
*Cla001815*	LOW QUALITY PROTEIN: salicylic acid-binding protein 2-like	Up	Up	Down	Up
*Cla016032*	scopoletin glucosyltransferase-like	Up	Down	Down	Down
*Cla017208*	cinnamoyl-CoA reductase 2-like	Up	Down	Up	Up
*Cla017207*	cinnamoyl-CoA reductase 2-like	Up	Down	Up	Up
**Environmental Information Procession**					
**Signal transduction**					
*Cla002572*	BTB/POZ and TAZ domain-containing protein 1	Down	Down	Up	Up
*Cla017611*	uncharacterized protein LOC103486691	Down	Down	Up	Up
*Cla016659*	two-component response regulator ARR12-like	Up	Up	Up	Up
*Cla008326*	putative cell division cycle ATPase	Up	Up	Down	Up
*Cla008169*	uncharacterized protein LOC101203772	Down	Down	Up	Up
*MSTRG.1055*	defensin-like protein 6	Up	Up	Up	Down
*Cla006543*	AT-hook motif nuclear-localized protein 15	Up	Up	Up	Up
**genetic information processing**					
**Folding, sorting and degradation**					
*Cla023118*	U-box domain-containing protein 26	Down	Down	Up	Up
**Mitochondrial biogenesis**					
*Cla009892*	mitochondrial translocator assembly and maintenance protein 41 homolog isoform X1	Down	Down	Up	Down
**Organismal Systems**					
**Environmental adaptation**					
*Cla007307*	WRKY70	Down	Down	Down	Up
*Cla007656*	probable WRKY transcription factor 31	Up	Up	Down	Down
*Cla018486*	probable receptor-like protein kinase At5g39030	Up	Down	Down	Down
*Cla007979*	probable transcription factor GLK2	Down	Down	Up	Up
***stress-related genes***					
*Cla023376*	Early nodulin-like protein 2 [Glycine soja]	Down	Down	Down	Up
*MSTRG.17433*	hypothetical protein Csa_6G059230	Up	Up	Down	Down
*Cla010283*	hypothetical protein Csa_2G237730	Down	Down	Up	Up
*Cla002904*	hypothetical protein Csa_3G822280	Down	Down	Up	Up
*Cla015065*	hypothetical protein Csa_7G397010	Down	Down	Down	Up
*Cla008418*	Iron-sulfur binding oxidoreductase	Up	Up	Up	Up
*Cla010875*	BAG family molecular chaperone regulator 6-like	Up	Down	Down	Down
*Cla012888*	classical arabinogalactan protein 10-like	Up	Up	Down	Up
*Cla013835*	formin-like protein 20-like	Up	Up	Down	Down
*Cla013527*	seed biotin-containing protein SBP65	Down	Down	Up	Up
*Cla004456*	uncharacterized protein LOC103491021	Down	Down	Up	Down
*Cla007700*	uncharacterized protein LOC103495564	Down	Down	Up	Up
*Cla011574*	14 kDaproline-rich protein DC2.15-like	Up	Up	Down	Down
*Cla018055*	probably inactive leucine-rich repeat receptor-like protein kinase At3g28040	Down	Down	Up	Up
*Cla019959*	universal stress protein A-like protein isoform X2	Down	Down	Up	Up
*Cla014570*	dehydrin DHN1-like	Down	Up	Up	Up
*Cla017570*	F-box/kelch-repeat protein At2g44130-like	Down	Down	Up	Up
*Cla009891*	haloacid dehalogenase-like hydrolase domain-containing protein 3	Down	Down	Up	Down
*Cla021693*	sugar transporter ERD6-like 16 isoform X2	Up	Down	Down	Down
*Cla011361*	bidirectional sugar transporter SWEET4-like	Down	Up	Down	Up
*MSTRG.15291*	no hit found	Down	Down	Up	Up
*MSTRG.20101*	no hit found	Down	Up	Up	Up
*MSTRG.967*	no hit found	Up	Up	Down	Down
*MSTRG.968*	no hit found	Up	Up	Down	Down

## Data Availability

The raw sequence data (in FastQ format) have been submitted to the NCBI SRA database (https://www.ncbi.nlm.nih.gov/sra/?term=. accessed on 19 January 2020) with the study accession (SRP243255). The plant materials are available from the corresponding author.

## Supplementary Materials

The following supporting information can be downloaded at: https://www.mdpi.com/article/10.3390/plants12040935/s1, Table S1: The integrated function of 22379 annotated genes. Table S2: The genes and annotation in module ‘lightpink4’. Table S3: The genes and annotation in module ‘lavenderblush1’. Table S4: Top 20 enriched in biological process by GO enrichment analyses of DEGs. Table S5: Top 20 enriched in cellular processes by GO enrichment analyses of DEGs. Table S6: Top 20 enriched in molecular functions by GO enrichment analyses of DEGs. Table S7: Summary of the RNA-Seq data. Figure S1: Sequencing saturation curves (a) and sequencing gene coverage analysis (b) of the 24 RNA-Seq samples. Figure S2: Weighted correlation network analysis of related genes response to lowlight stress of watermelon fruit expansion. (a) Heriarchical clustering tree showing co-expression modules. Each leaf in the tree is one gene. The major tree branches constitute 10 modules labeled by different colors. (b) Module-trait relationship. The left lane indicates 10 module eigengenes. The right lane indicates the module-trait correlation from − 1 to 1. FVD: fruit vertical diameter; FTD: fruit trans-verse diameter; SFW: single fruit weight; SS: soluble sugar; AA: amino acid. Figure S3: Functional classifications of DEGs in watermelon fruit flesh using WEGO. The *X*-axis shows the GO functional categories of cellular components, molecular functions and biological processes. The left *Y*-axis shows the percentage of each category; the right *Y*-axis shows the number of DEGs in each category. Figure S4: TF families of the DEGs at 0 DAP (a), 3 DAP (b), 9 DAP (c), and 15 DAP (d) of watermelon fruit expansion under low light stress. A total of 97, 277, 143 and 419 TFs were differentially expressed at 0 DAP, 3 DAP, 9 DAP and 15 DAP after low light stress, respectively, and the percentages of TF families in DEGs and all genes in watermelon genome were shown as blue bars and red bars (P < 0.01 and FDR < 0.05), respectively. Figure S5: The raw FPKM values of TF DEGs were first normalized by logarithmic method and a heat map was constructed to show the different expression profiles by the Heml software (version 1.0, http://hemi.biocuckoo.org/. accessed on 9 July 2020). *X*-axis, samples; *Y*-axis, differentially expressed gene names. (a) BHLH, (b) ERF, (c) NAC, (d) WRKY.
