# Supplementary material for: Transcriptome Analysis Reveals the Genes Related to Water-Melon Fruit Expansion under Low-Light Stress
Source: Plants (Basel). 2023 Feb 18;12(4):935. doi: 10.3390/plants12040935 (PMC9958833; doi:10.3390/plants12040935)

Figure S1. Sequencing saturation curves (a) and sequencing gene coverage analysis (b) of the 24 RNA-Seq samples.

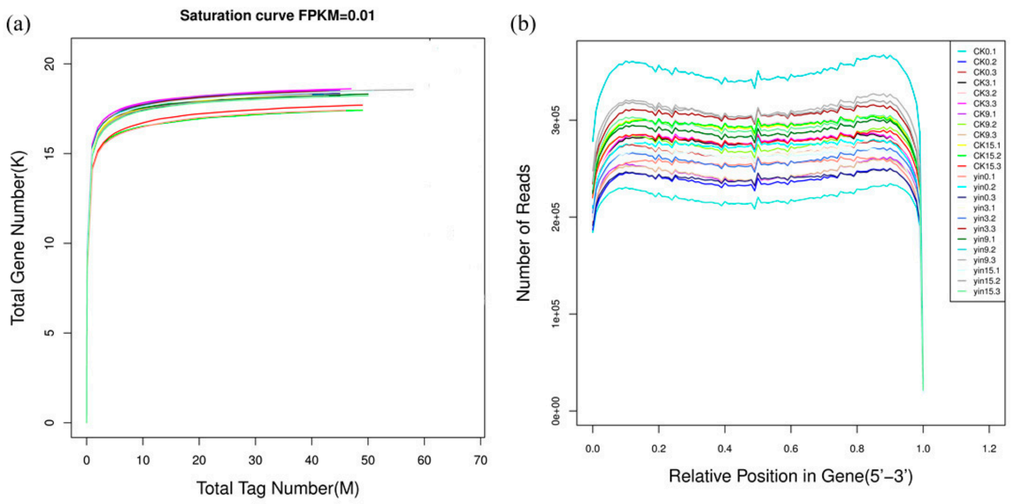

Supplement: Supplementary file 1 [file plants-12-00935-s001.zip › Figure S1 Sequencing saturation curves (a) and sequencing gene coverage analysis (b) of the 24 RNA-Seq samples.pdf]
