# Supplementary material for: Transcriptome Analysis Reveals the Genes Related to Water-Melon Fruit Expansion under Low-Light Stress
Source: Plants (Basel). 2023 Feb 18;12(4):935. doi: 10.3390/plants12040935 (PMC9958833; doi:10.3390/plants12040935)

Figure S3. Functional classifications of DEGs in watermelon fruit flesh using WEGO. The x-axis shows the GO functional categories of cellular components, molecular functions and biological processes. The left y-axis shows the percentage of each category; the right y-axis shows the number of DEGs in each category.

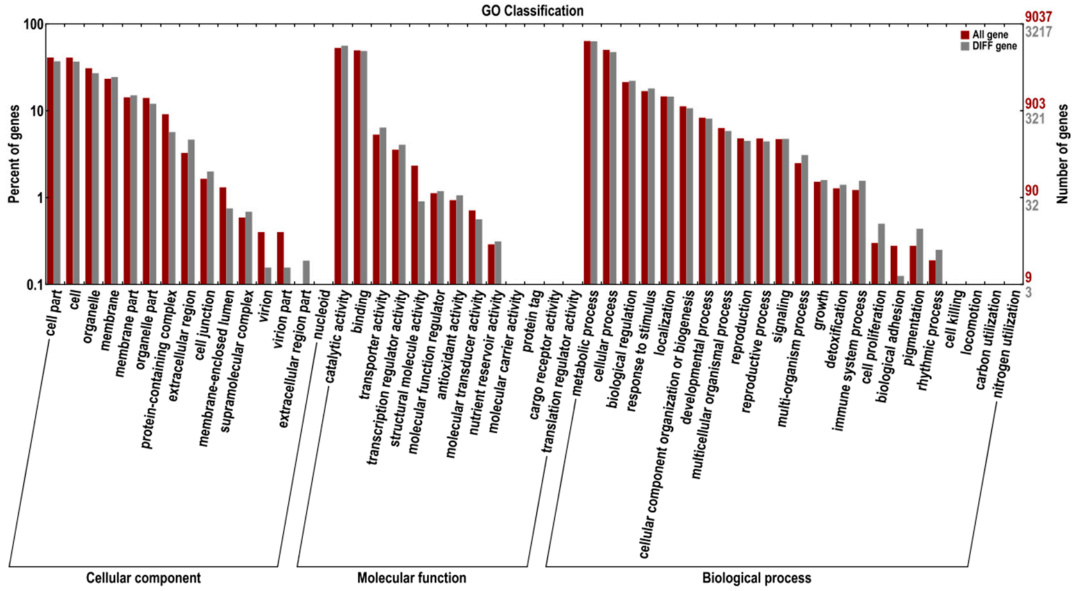

Supplement: Supplementary file 1 [file plants-12-00935-s001.zip › Figure S3 Functional classifications of DEGs in watermelon fruit flesh using WEGO.pdf]
