# Supplementary material for: Transcriptome Analysis Reveals the Genes Related to Water-Melon Fruit Expansion under Low-Light Stress
Source: Plants (Basel). 2023 Feb 18;12(4):935. doi: 10.3390/plants12040935 (PMC9958833; doi:10.3390/plants12040935)
Supplement: Supplementary file 1 [file plants-12-00935-s001.zip › Table S4 Top 20 enriched in biological process by GO enrichment analyses of DEGs.pdf]

Table S2. Top 20 enriched in biological process by GO enrichment analyses of DEGs

GO.ID	Term	Annotated	Significant	Expected	KS
GO:0055114	oxidation-reduction process	999	433	357.97	1.10E-07
GO:0009863	salicylic acid mediated signaling pathway	49	26	17.56	0.00032
GO:0071555	cell wall organization	195	93	69.87	0.00054
GO:0006261	DNA-dependent DNA replication	50	30	17.92	0.00099
GO:0046165	alcohol biosynthetic process	99	51	35.47	0.00106
GO:0055085	transmembrane transport	476	200	170.56	0.00192
GO:0010103	stomatal complex morphogenesis	31	18	11.11	0.00241
GO:0009753	response to jasmonic acid	53	28	18.99	0.00246
GO:0005991	trehalose metabolic process	10	8	3.58	0.0028
GO:0006011	UDP-glucose metabolic process	6	5	2.15	0.00302
GO:0010583	response to cyclopentenone	20	14	7.17	0.00314
GO:0055067	monovalent inorganic cation homeostasis	7	6	2.51	0.00336
GO:0030245	cellulose catabolic process	11	9	3.94	0.00343
GO:0090332	stomatal closure	7	4	2.51	0.00363
GO:0006541	glutamine metabolic process	17	12	6.09	0.00432
GO:0005976	polysaccharide metabolic process	285	131	102.12	0.00503
GO:0051301	cell division	224	87	80.27	0.00511
GO:0045454	cell redox homeostasis	67	34	24.01	0.00522
GO:0046351	disaccharide biosynthetic process	12	8	4.3	0.006
