# Supplementary material for: Transcriptome Analysis Reveals the Genes Related to Water-Melon Fruit Expansion under Low-Light Stress
Source: Plants (Basel). 2023 Feb 18;12(4):935. doi: 10.3390/plants12040935 (PMC9958833; doi:10.3390/plants12040935)
Supplement: Supplementary file 1 [file plants-12-00935-s001.zip › Table S5 Top 20 enriched in cellular processes by GO enrichment analyses of DEGs.pdf]

Table S3. Top 20 enriched in cellular processes by GO enrichment analyses of DEGs

GO.ID	Term	Annotated	Significant	Expected	KS
GO:0016021	integral component of membrane	1095	436	379.34	5.70E-07
GO:0005576	extracellular region	297	151	102.89	4.60E-05
GO:0048046	apoplast	91	52	31.52	5.50E-05
GO:0009505	plant-type cell wall	56	33	19.4	0.00084
GO:0009570	chloroplast stroma	198	94	68.59	0.00194
GO:0005886	plasma membrane	507	200	175.64	0.00722
GO:0044421	extracellular region part	10	8	3.46	0.00788
GO:0010287	plastoglobule	17	11	5.89	0.00879
GO:0009506	plasmodesma	148	64	51.27	0.00923
GO:0044815	DNA packaging complex	35	20	12.12	0.01361
GO:0010598	NAD(P)H dehydrogenase complex (plastoqui...	6	5	2.08	0.01874
GO:0005839	proteasome core complex	11	8	3.81	0.02335
GO:0071944	cell periphery	646	268	223.79	0.03
GO:0000786	nucleosome	33	18	11.43	0.03527
GO:1990104	DNA bending complex	33	18	11.43	0.03527
GO:0032993	protein-DNA complex	46	23	15.94	0.03904
GO:0005795	Golgi stack	15	6	5.2	0.03919
GO:0005875	microtubule associated complex	47	23	16.28	0.04283
GO:0044430	cytoskeletal part	89	35	30.83	0.04296
GO:0030312	external encapsulating structure	160	80	55.43	0.04715
