# Supplementary material for: Transcriptome Analysis Reveals the Genes Related to Water-Melon Fruit Expansion under Low-Light Stress
Source: Plants (Basel). 2023 Feb 18;12(4):935. doi: 10.3390/plants12040935 (PMC9958833; doi:10.3390/plants12040935)
Supplement: Supplementary file 1 [file plants-12-00935-s001.zip › Table S6 Top 20 enriched in molecular functions by GO enrichment analyses of DEGs.pdf]

Table S4. Top 20 enriched in molecular functions by GO enrichment analyses of DEGs

GO.ID	Term	Annotated	Significant	Expected	KS
GO:0016760	cellulose synthase (UDP-forming) activit...	24	17	8.8	9.90E-05
GO:0016705	oxidoreductase activity, acting on paire...	203	104	74.43	0.00018
GO:0016491	oxidoreductase activity	970	434	355.63	0.00037
GO:0005544	calcium-dependent phospholipid binding	10	8	3.67	0.00079
GO:0016709	oxidoreductase activity, acting on paire...	46	23	16.86	0.0009
GO:0005506	iron ion binding	153	78	56.09	0.00107
GO:0051753	mannan synthase activity	6	6	2.2	0.0013
GO:0015291	secondary active transmembrane transport...	108	49	39.6	0.00189
GO:0051213	dioxygenase activity	68	36	24.93	0.00233
GO:0004364	glutathione transferase activity	6	6	2.2	0.00285
GO:0046983	protein dimerization activity	232	110	85.06	0.00292
GO:0035251	UDP-glucosyltransferase activity	62	41	22.73	0.00338
GO:0008810	cellulase activity	11	9	4.03	0.00393
GO:0016757	transferase activity, transferring glyco...	320	153	117.32	0.00463
GO:0010333	terpene synthase activity	8	4	2.93	0.0048
GO:0031406	carboxylic acid binding	44	25	16.13	0.00498
GO:0030414	peptidase inhibitor activity	10	7	3.67	0.00708
GO:0045548	phenylalanine ammonia-lyase activity	11	8	4.03	0.00719
GO:0008289	lipid binding	67	31	24.56	0.00733
GO:0052689	carboxylic ester hydrolase activity	96	42	35.2	0.0075
