# Supplementary figures and images for: Transcriptome Analysis Reveals the Genes Related to Water-Melon Fruit Expansion under Low-Light Stress
Source: Plants (Basel). 2023 Feb 18;12(4):935. doi: 10.3390/plants12040935 (PMC9958833; doi:10.3390/plants12040935)

# Cluster Dendrogram

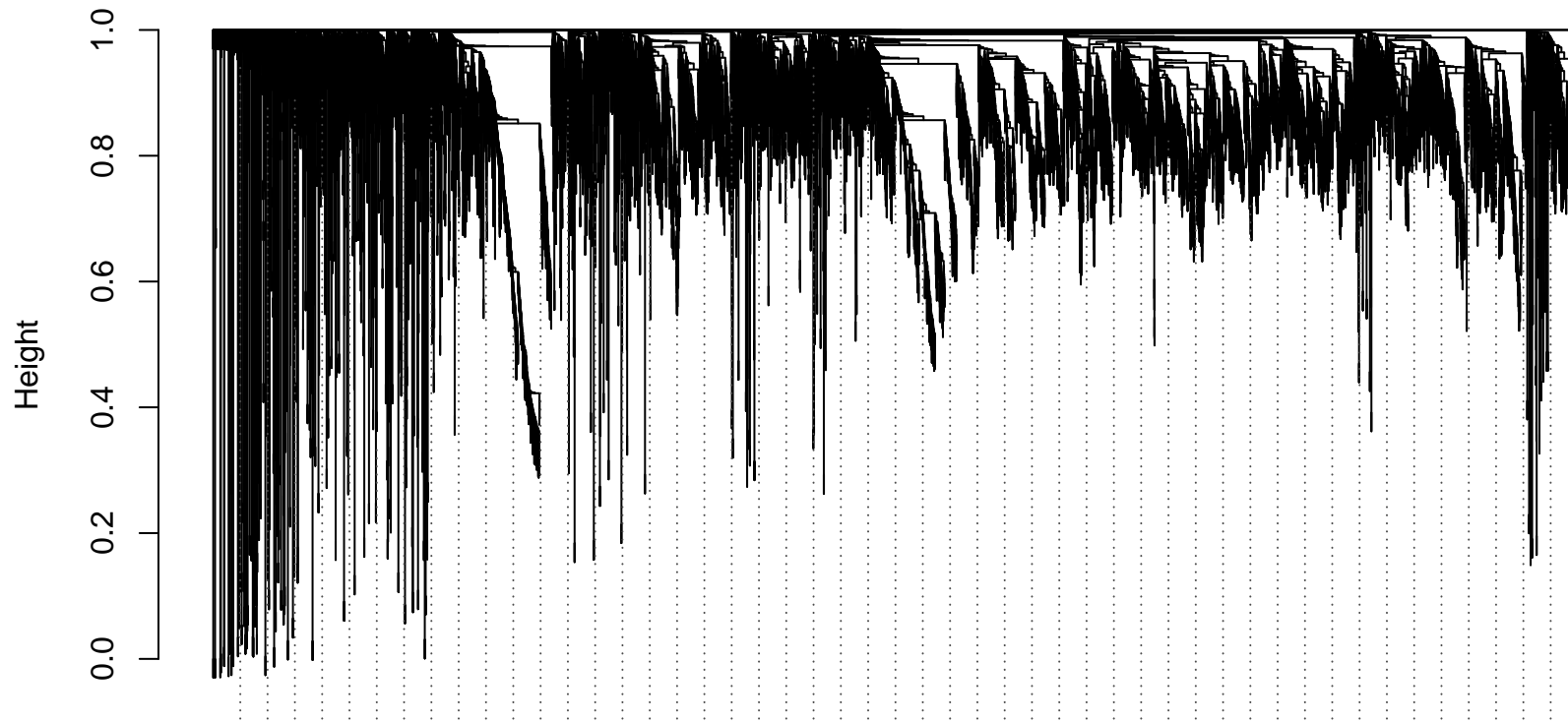

Dynamic Tree Cut

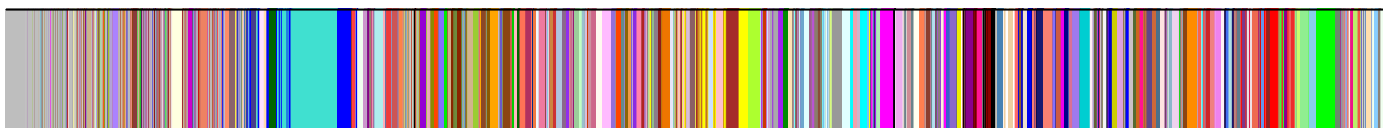

Merged dynamic

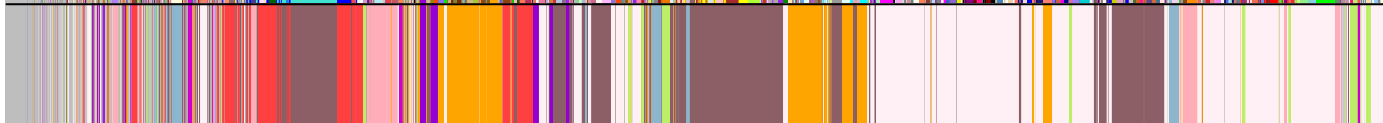

Supplement: Supplementary file 1 [file plants-12-00935-s001.zip › Figure S2a Weighted correlation network analysis of related genes response to lowlight stress of watermelon fruit expansion.pdf]

# Module–trait relationships

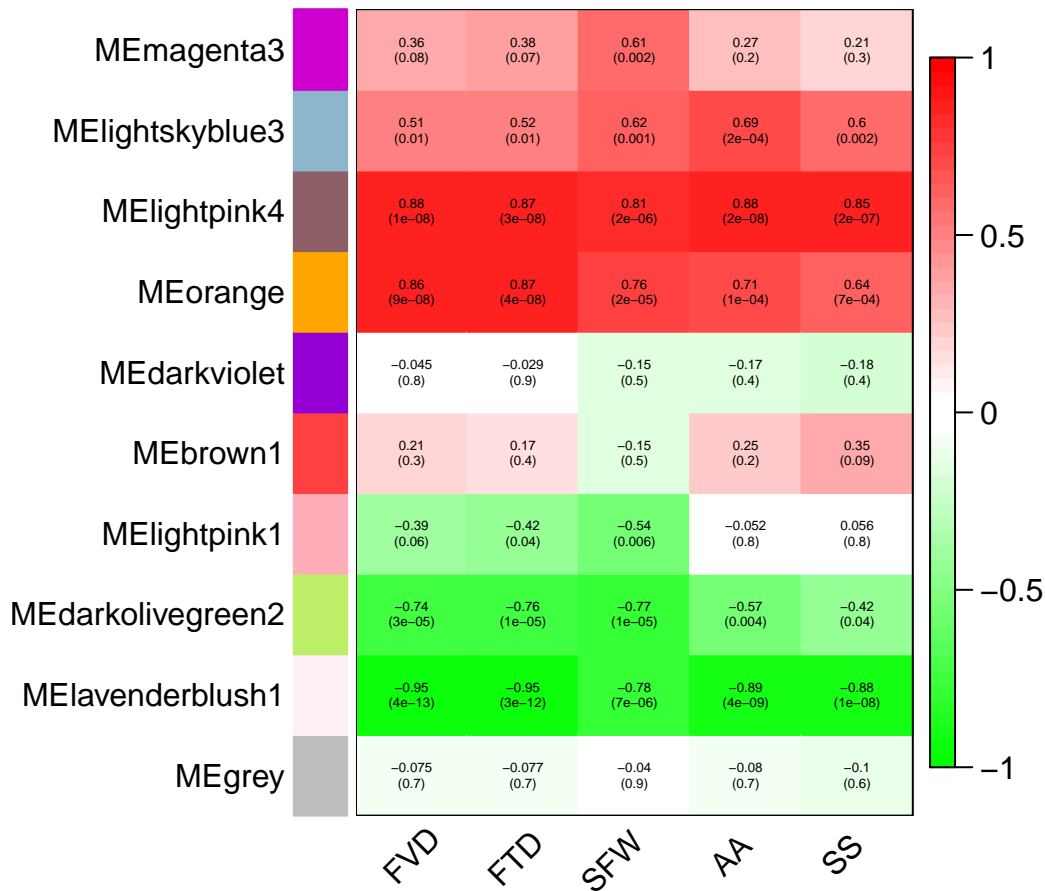

Supplement: Supplementary file 1 [file plants-12-00935-s001.zip › Figure S2b Weighted correlation network analysis of related genes response to lowlight stress of watermelon fruit expansion.pdf]

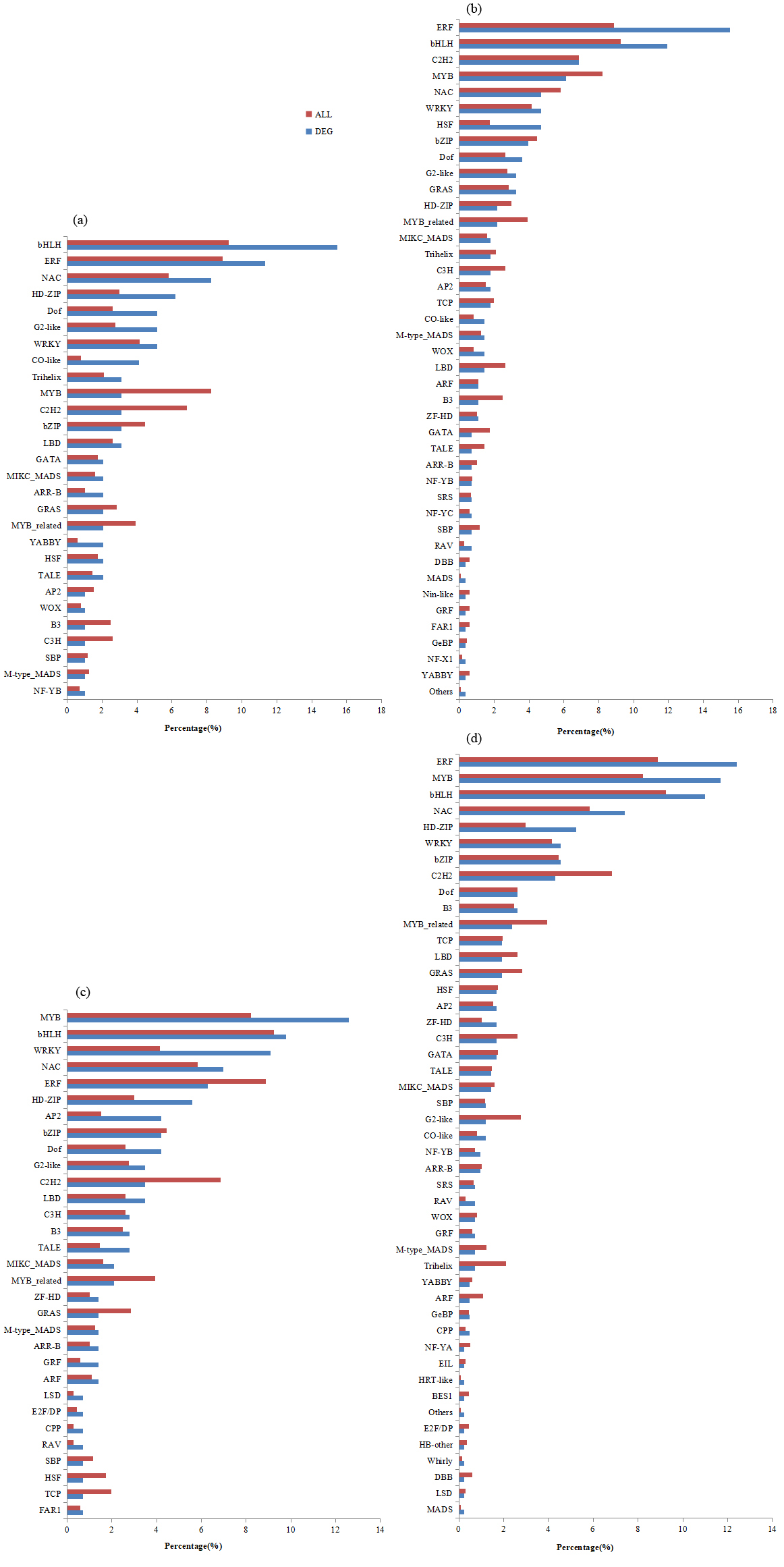

Supplement: Supplementary file 1 [file plants-12-00935-s001.zip › Figure S4 TF families of the DEGs at 0 DAP (a), 3 DAP (b), 9 DAP (c), and 15 DAP (d) of watermelon fruit expansion under low light stress..jpg]

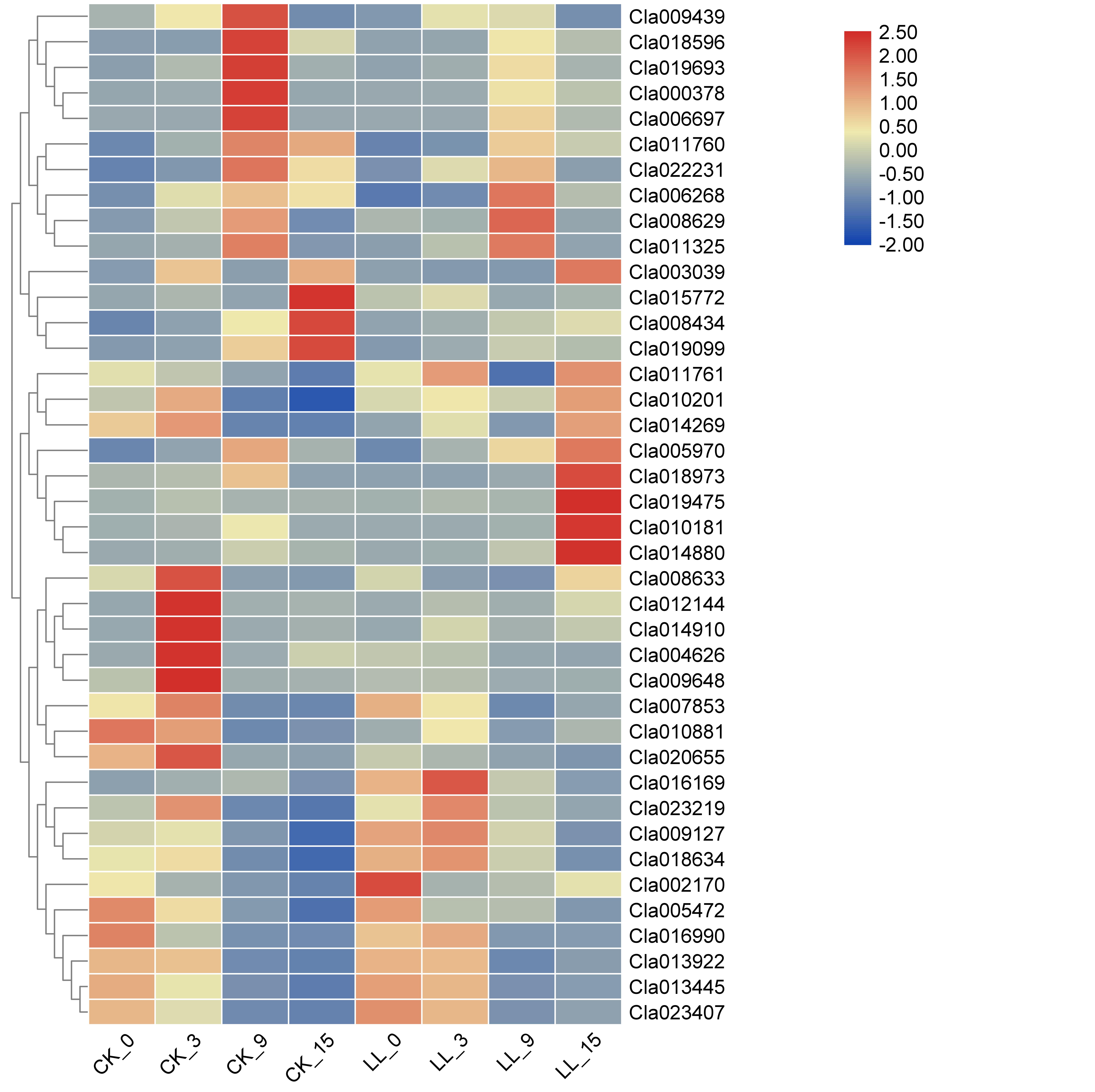

Supplement: Supplementary file 1 [file plants-12-00935-s001.zip › Figure S5 c The raw FPKM values of TF DEGs were first normalized by logarithmic method and a heat map was constructed to show the different expression profiles -NAC.jpg]

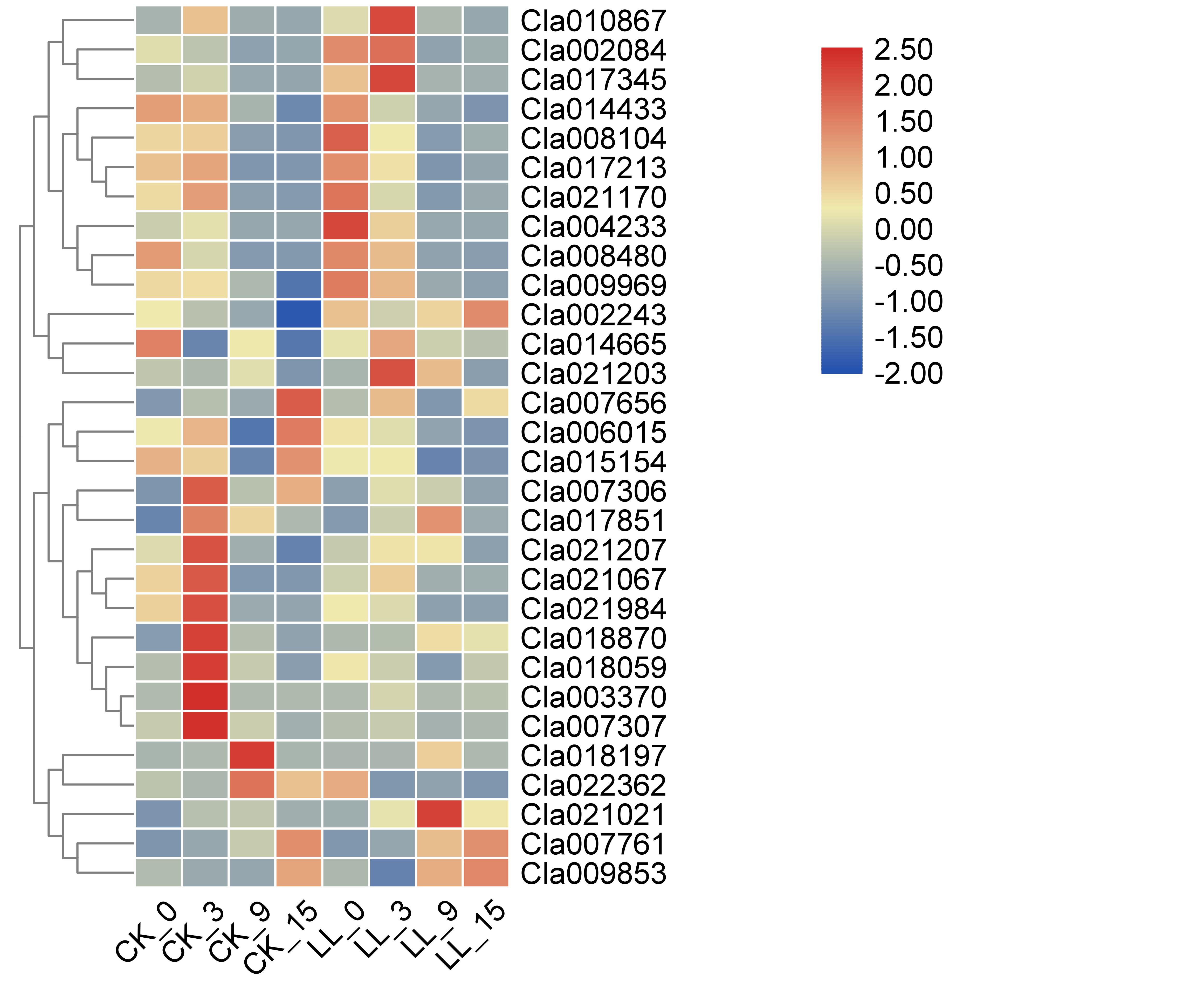

Supplement: Supplementary file 1 [file plants-12-00935-s001.zip › Figure S5 d The raw FPKM values of TF DEGs were first normalized by logarithmic method and a heat map was constructed to show the different expression profiles -WRKY.jpg]
